# Research on the mechanism and application of wedge cutting blasting with hole-inner delay

**DOI:** 10.1038/s41598-024-62318-2

**Published:** 2024-05-18

**Authors:** Bing Cheng, Quan Wang, Haibo Wang, Qi Zong, Pengfei Gao

**Affiliations:** 1 Joint National-Local Engineering Research Centre for Safe and Precise Coal Mining, Anhui University of Science and Technology, Huainan, 232001 China; 2 State Key Laboratory of Mining Response and Disaster Prevention and Control in Deep Coal Mines, Anhui University of Science and Technology, Huainan, 232001 China; 3 Anhui Engineering Laboratory of Explosive Materials and Technology, Anhui University of Science and Technology, Huainan, 232001 China; 4 School of Chemical and Blasting Engineering, Anhui University of Science and Technology, Huainan, 232001 China; 5 School of Civil Engineering and Architecture, Anhui University of Science and Technology, Huainan, 232001 China

**Keywords:** Wedge cutting blasting, Hole-inner delay, Blasting mechanism, Simulation, Application, Civil engineering, Engineering

## Abstract

To increase the efficiency of deep-hole blasting driving in mine rock tunnels, an innovative pattern of wedge cutting blasting with hole-inner delay was proposed. First, the blasting mechanisms of conventional and innovative wedge cutting patterns were theoretically investigated. The results showed that the resistance from large upper rock blocks and the clamping action from the surrounding rock were the major challenges of conventional cutting methods. For the innovative cutting pattern, under the conversion of the spatial distribution and release sequence of blasting energy, the first blasting of the upper charge can strengthen the breaking of the upper rock mass and create a new free surface, which provides favorable conditions for the delayed blasting of the bottom charge. Second, finite element models of two cutting patterns were established and solved, and the simulation results visually revealed the propagation of a stress wave. Critically, the stress strength in the upper cavity increased by 66–83% under the action of the upper charge, which was conducive to the breaking of the upper rock mass and the generation of a new free surface. Therefore, the rock mass in the bottom cavity can be readily broken and discharged. Ultimately, field applications were executed in a rock tunnel. Compared with a conventional cutting pattern, the proposed innovative cutting pattern can prominently increase the cycle advance and hole utilization and greatly reduce the unit consumption of explosives and detonators. This research confirms the usability of the innovative wedge cutting pattern with hole-inner delay in deep-hole blasting driving of rock tunnels.

## Introduction

**Figure 1:**
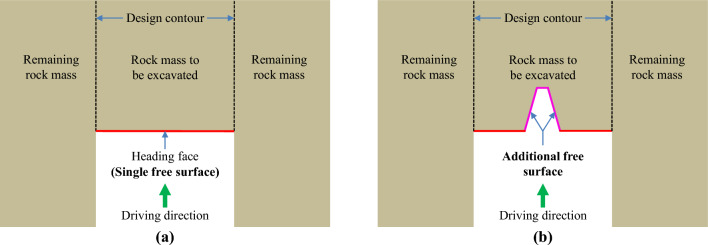
Diagram of the free surface of rock tunnel blasting excavation: (**a**) single free surface before cutting blasting and (**b**) additional free surface created by cutting blasting.

Engineering blasting techniques use the substantial amount of energy released by explosive detonation to destroy rock masses, concrete, and other solid materials to achieve specific engineering purposes. In view of the distinct properties of simple technical processes, high geological adaptability, and reasonable capital investment, these techniques are commonly employed in various mine construction projects, particularly the driving of underground rock tunnels^1–4^. As indicated in Fig. 1a, in the blasting driving process of a rock tunnel, only the heading face is established as the single free surface for broken rock bulking and rock block movement. Therefore, the powerful clamping action from the surrounding rock increases the blasting difficulty and reduces the driving efficiency. Fortunately, as the first step of full-section blasting driving, cutting blasting can create an additional free surface for subsequent blasting steps^5–8^, thus dramatically weakening the clamping action from the surrounding rock, as presented in Fig. 1b. In addition, an appropriate cutting pattern is crucial for achieving acceptable rock breaking results and satisfactory driving efficiency during blasting work in tunnel driving.

Currently, the most frequently used methods for the rock tunnel cutting blasting are classified into vertical hole and oblique hole cutting modes according to the angle between the cutting hole and the face. The most common oblique hole cutting mode is based on the wedge cutting blasting method^9–12^. Sustainable construction practices have proven that the wedge cutting blasting method possesses remarkable strengths, such as lower energy consumption, larger cavity size, and fewer blasting holes, over the vertical hole cutting method^13,14^. Therefore, wedge cutting blasting has evolved into the most widely employed cutting method in the blasting driving construction of large section tunnels. To determine the high-quality cutting blasting effect, several studies have been carried out on the rock breaking mechanism, optimal design, and practical application of wedge cutting patterns, and fruitful research findings have been achieved. For instance, Shapiro^15^ experimentally compared the overall driving efficiency under different cutting blasting patterns and concluded that wedge cutting blasting was the most effective cutting mode for underground rock tunnel driving. Wang et al.^16^ theoretically revealed the failure mechanism of a rock mass in a wedge cutting cavity under the dynamic conditions of a stress wave and detonation gas and achieved an excellent application effect in a driving project involving a large section of a rock tunnel. Based on the vibration signals obtained from blasting sites, Man et al.^17^ analytically investigated the frequency features of blasting energy under different cutting patterns. These researchers revealed that the blasting energy of the wedge cutting pattern was mostly distributed in the high-frequency region, while that of the vertical hole cutting pattern was mostly concentrated in the low-frequency region. Pu et al.^18^ and Xiong et al.^19^ systematically studied the influencing factors of wedge cutting patterns by using correlation degree theory and an analytic hierarchy process, respectively, and confirmed that the angle of the cutting holes was critical for wedge cutting blasting. Through similar material model experiments, Yang et al.^20^ researched the effect of the cutting hole angle on the rock block size and cavity morphology parameters of wedge cutting blasting and further provided a rational range of cutting hole angles. Gao et al.^21^ numerically studied the influence law of the initiation mode of the cutting holes on the damage distribution within the cutting cavity. They concluded that the staggered arrangement of the initiation points in the left and right cutting holes was the most reasonable mode. Hu et al.^22^ generated a solid model of wedge cutting patterns and simulated the forming process of cutting cavities by defining the rock strength as the element failure threshold. Chen et al.^23^ carried out numerical analyses and model experiments of wedge cutting blasting under unidirectional pressure and described the adverse effects of unidirectional pressure on damage evolution, cavity volume, and block distribution. Using numerical simulation and full-scale tests, Cheng et al.^24^ and Gao et al.^25^ investigated the effect of the charge diameter on hard rock wedge cutting blasting and concluded that increasing the charge diameter could aggravate the breaking degree of the rock mass. In addition to the above studies that have focused on traditional wedge cutting patterns, several recent studies have introduced a hole-outer delay into deep-hole wedge cutting blasting. For example, Shan et al.^26^ and Lou et al.^27^ proposed a modified mode of wedge cutting blasting with the delayed detonation of auxiliary vertical holes and reported that the delayed detonation of auxiliary vertical holes was beneficial for eliminating the residual rock mass in the bottom range to some extent. Through numerical simulation and theoretical derivation, Cheng et al.^28^ and Ding et al.^29^ described the rock breaking principle of double-wedge cutting blasting with hole-outer delay and subsequently verified the engineering applicability of this cutting pattern in deep-hole blasting driving. Moreover, Zhang et al.^30^ added several straight holes detonated simultaneously with shallow cutting holes into a double wedge cutting pattern, which could cause preliminary damage to the bottom rock during hard rock cutting blasting. However, these improved wedge cutting patterns using the hole-outer delay have achieved only limited success in increasing the deep-hole cutting efficiency and have led to additional problems such as complex hole arrangements and difficult drilling operations. Based on the above review of the aforementioned literature, most existing studies have focused on optimizing the design of conventional wedge cutting blasting, and a few studies have involved wedge cutting blasting with hole-outer delay; however, no research has been conducted on wedge cutting blasting with hole-inner delay.

With the rapid development and progress of the national economy, there is a growing need for various mineral resources, such as coal and metal ore. For mineral resources stored underground, the fast speed of rock tunnel driving is a necessary condition for high mining efficiency. Nevertheless, in most rock tunnel blasting projects in China, blasting holes less than 2.0 m in length have been widely adopted to obtain a relatively short driving footage of 1.6–1.8 m, which can lead to an imbalance between mining and driving^31^. In recent years, to address the low efficiency of rock tunnel blasting driving, several researchers and engineers have proposed a novel technique of deep-hole blasting driving with a hole depth greater than 2.5 m^32^. However, a new challenge has arisen in that the clamping action can become significantly stronger with increasing hole depth, which can cause serious obstacles to the expulsion of the bottom rock mass. Predictably, an unsatisfactory cutting effect can negatively impact the overall rock breaking results and blasting cycle advance. Without a high cutting blasting efficiency, the deep-hole blasting technique cannot effectively guarantee a balance between mining and driving.

As a result, to achieve highly efficient deep-hole cutting blasting in mine rock tunnel driving, an innovative pattern of wedge cutting blasting using hole-inner delay was developed in the present study. First, the rock breaking and cavity forming mechanisms of conventional and innovative wedge cutting patterns were theoretically explained. Subsequently, the propagation of stress waves and the distribution features of blasting stress in the cutting cavity were presented explicitly through finite element simulation, and then the rock failure mechanism of the innovative cutting pattern was further revealed based on the simulation results. Finally, field applications of deep-hole blasting driving were performed in a rock tunnel to explore the practicability of the innovative wedge cutting pattern.

## Theoretical analysis of the blasting mechanism

### Conventional pattern of wedge cutting blasting

**Figure 2:**
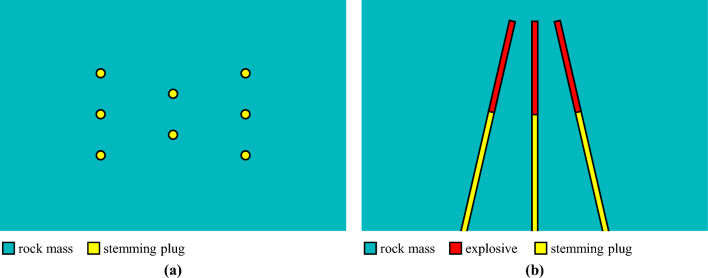
Diagram of the conventional wedge cutting pattern: (**a**) primary view and (**b**) overhead view.

In conventional wedge cutting blasting, two rows of blasting holes are arranged symmetrically and obliquely, and a wedge-shaped cavity is generated after charge blasting. However, when hard rock is encountered during rock tunnel blasting driving, the blasting energy from the cutting holes becomes insufficient. Some researchers have suggested increasing the blasting energy and enhancing rock damage by adding several vertically charged holes in the middle zone, which are detonated simultaneously with oblique cutting holes, as shown in Fig. 2. With the aid of the extra blasting energy from the vertically charged holes, the hard rock mass within the cutting cavity can be adequately broken up and thrown out. Since the blasting energy is mainly concentrated in the bottom section, this type of wedge cutting blasting is also referred to as energy-concentrated wedge cutting blasting^33^.

Many studies have demonstrated that conventional wedge cutting blasting can achieve an acceptable rock breaking effect and high hole utilization in shallow hole blasting driving, but obtaining a cutting cavity that conforms to the design depth in deep-hole blasting driving is challenging. The main factors contributing to the latter phenomenon are as follows: First, with increasing depth of the blasting holes, the explosives are more concentrated in the bottom section, and those in the upper uncharged section are farther from the blasting charge. Understandably, it is difficult to achieve effective damage and full ejection of the rock mass located in the upper uncharged section, which resists the expulsion of the rock mass in the bottom section. Second, since only the heading face is used as the free surface, the increase in hole depth can significantly enhance the surrounding rock clamping action on the rock mass in the bottom section. When the surrounding rock clamping action exceeds the throwing capability of blasting charge, it is difficult to eject the rock mass in the bottom section, so it basically remains in its original position. In summary, the two main problems of conventional cutting patterns in deep-hole blasting driving are the resistance from large upper rock blocks and the clamping action from the surrounding rock.

### Innovative pattern of wedge cutting blasting

**Figure 3:**
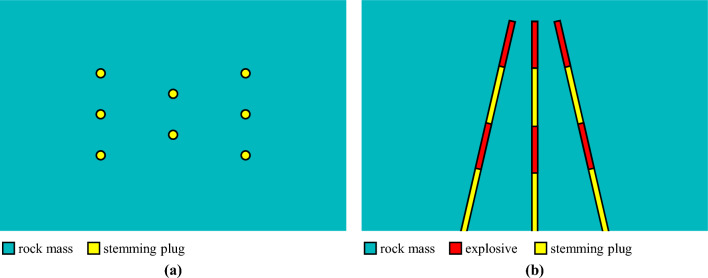
Diagram of the innovative wedge cutting pattern: (**a**) primary view and (**b**) overhead view.

According to the above description of conventional wedge cutting patterns, how to achieve the high-efficiency wedge cutting blasting during deep-hole blasting driving is an urgent problem that needs to be solved. Therefore, in the present paper, the principle of hole-inner delay is introduced to deep-hole wedge cutting blasting to develop an innovative cutting blasting pattern. As exhibited in Fig. 3, in the specific design scheme, the charge in each blasting hole is divided into upper and bottom parts without changing the hole arrangement, and compared with that of the upper charge, the detonation time of the bottom charge is delayed.

**Figure 4:**
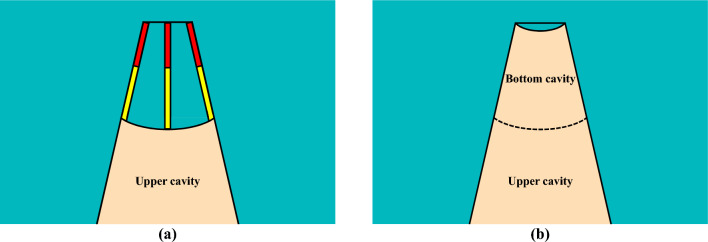
Blasting process of the innovative wedge cutting pattern: (**a**) first blasting of the upper charge and (**b**) delayed blasting of the bottom charge.

For the proposed innovative cutting blasting pattern, the entire process of rock breaking and throwing is separated into two steps using the hole-inner delay. After the first blasting of the upper charge, the rock mass in the upper section is broken into small rock blocks under the strong stress loading of the upper charge. There is a short distance in the uncharged section from the upper charge to the heading face; thus, only few large rock blocks can be produced in this section. Due to the shallow cutting depth of the upper charge section, the surrounding rock clamping action did not exceed the blasting throwing capacity. Therefore, the rock blocks in the upper cutting cavity can be fully thrown out under the detonation gas of the upper charge. As displayed in Fig. 4a, a new free surface was induced at this time, which means that the clamping force borne by the bottom rock is significantly reduced.

Similarly, after the delayed blasting of the bottom charge, the rock mass in the bottom section can be broken into small rock blocks under the strong stress loading of the bottom charge. There is a short distance of the uncharged section from the bottom charge to the new surface; thus, few large rock blocks are generated in this section. Then, because the clamping action exerted on the bottom rock is reduced, the surrounding rock clamping action does not exceed the blasting throwing capacity in the bottom section. Because of the weak clamping action, the rock blocks in the bottom cutting cavity are bound to be fully ejected under the detonation gas of the bottom charge. Consequently, a cutting cavity that basically conforms to the design depth can be achieved, as shown in Fig. 4b.

In summary, the innovative pattern of wedge cutting blasting involves the conversion of the spatial distribution and release sequence of blasting energy through the use of the hole-inner delay. From a spatial perspective, a relatively uniform distribution of blasting energy can prevent the formation of large rock blocks in the upper part of the cutting cavity, thus eliminating the resistance to the expulsion of the bottom rock mass. From a temporal perspective, the first blasting of the upper charge can create a new free surface for the delayed blasting of the bottom charge, thus reducing the clamping action exerted on the bottom rock mass. Under the favorable conditions of time and space generated by the hole-inner delay, the rock mass in the cutting cavity can be effectively destroyed and fully removed.

## Finite element simulation

### Finite element model

**Figure 5:**
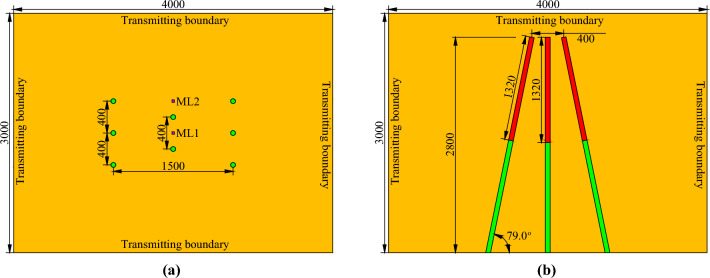
Geometric parameters of the conventional wedge cutting model (unit: mm): (**a**) primary view and (**b**) overhead view.

**Figure 6:**
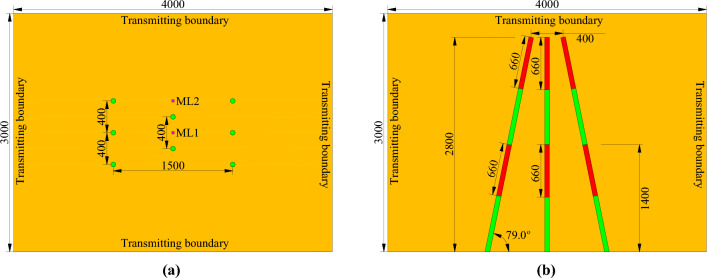
Geometric parameters of the innovative wedge cutting model (unit: mm): (**a**) primary view and (**b**) overhead view.

To date, finite element simulations have been rapidly developed and widely used in engineering fields, especially in solving problems related to engineering blasting^34–36^. In this study, finite element models of conventional and innovative wedge cutting patterns were established by using eight node solid elements in the ANSYS program. The geometric parameters of the two finite element models were taken from the subsequent blasting experiments and are presented in Figs. 5 and 6.

The commonalities between the two finite element models lie in the model size and hole arrangement. The length, width, and height of the rock mass model were 4000, 3000, and 3000 mm, respectively. Six oblique cutting holes with an angle of 79.0° were symmetrically fixed on the left and right sides, and the spacing, top spacing, bottom spacing, and vertical depth of the oblique cutting holes were 400, 1500, 400, and 2800 mm, respectively. Two vertical cutting holes were fixed at the center of the cutting cavity, and the spacing and depth of the vertical cutting holes were 400 mm and 2800 mm, respectively. The differences between the two finite element models lie in the charge structure and the initiation time. For the conventional wedge cutting model, all blasting holes were continuously charged with a charge length of 1320 mm. Moreover, the explosive in each blasting hole was detonated at 0 μs. For the innovative wedge cutting model, the blasting charge in each blasting hole was separated into upper and bottom parts, and the lengths of the upper bottom charges were both 660 mm. The cutting depths corresponding to the upper and bottom charges were 1400 and 2800 mm, respectively. Furthermore, by using the keyword *INITIAL_DETONATION, the detonation times of the upper and bottom charges were set as 0 and 500 μs, respectively.

According to the phase state in the blasting process, Lagrangian elements were used to simulate the rock mass and stemming plug viewed as solid materials, while arbitrary Lagrangian Euler (ALE) elements were employed to describe the explosive and air considered to be fluid materials^37^. The interaction between the rock mass and stemming plug was handled by the automatic contact algorithm, and the explosive and air were bound together into a multimaterial group without defining a special algorithm. Critically, the transfer of mechanical information between the two phases of materials was realized by setting the fluid‒solid interaction in the keyword *CONSTRAINED_LAGRANGE_IN_SOLID^38,39^. Moreover, in addition to the heading face used as a free surface, transmitting boundary conditions were set on the other surfaces to prevent the adverse impact of boundary reflection on the numerical precision of the simulation results^40^. The configuration of the transmitting boundary necessitated the use of the keywords *Non_Reflecting_Boundary and *SET_SEGMENT.

**Figure 7:**
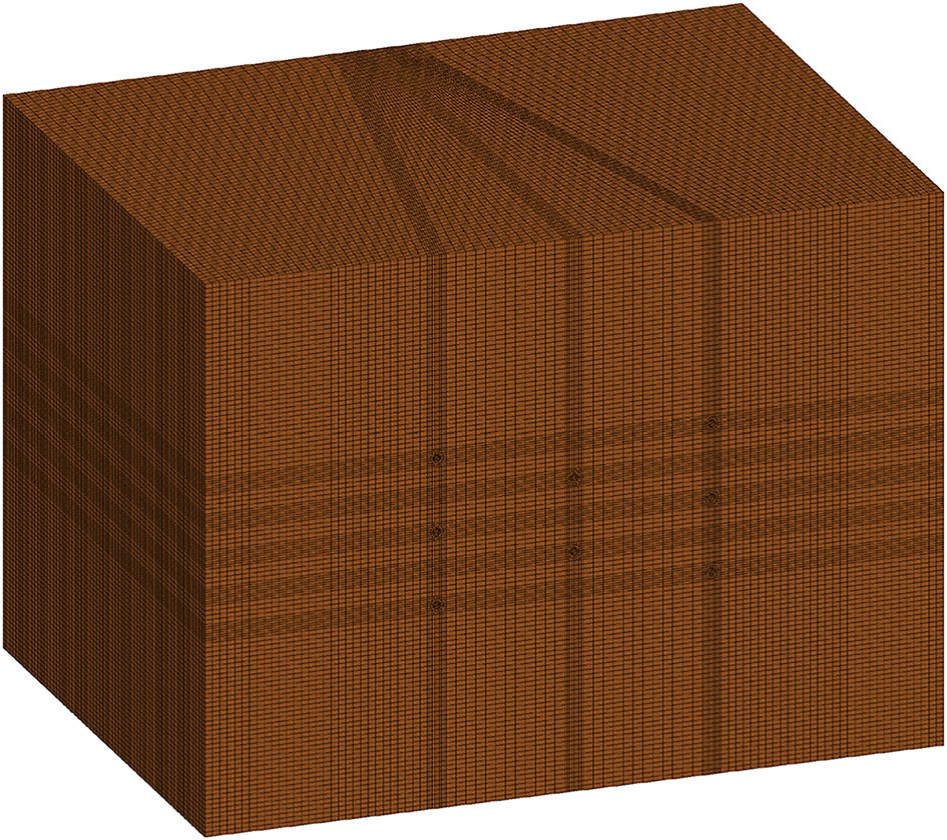
Element generation of the rock mass model.

In addition, previous research^41–44^ revealed that the calculation precision and numerical reliability of the simulation results largely depend on the element size. Therefore, it was extremely important to perform tentative simulations to acquire an optimal element size and thus avoid severe distortion. To achieve this purpose, the peak stress at specific locations was selected as the evaluation criterion for the simulation results. Then, the element size was gradually reduced; that is, the element amount was continually increased, until the discrepancy between the two adjacent simulations was lower than 5%^45^. Through the above efforts, the element amount of the rock mass model was determined to be 1,831,296. The element generation of the rock mass model is shown in Fig. 7.

### Constitutive models and materials parameters

In this simulation, four materials were employed, including an explosive, air, a rock mass, and a stemming plug. Notably, the dynamic behaviors of solid media can be described by constitutive models, while descriptions of fluid media require the use of both constitutive models and state equations. Therefore, the constitutive model of *MAT_HIGH_EXPLOSIVE_BURN was used for the explosive, and the complex relationship among the pressure, energy, and volume was represented by the JWL state equation^46–48^. Similarly, the *MAT_NULL constitutive model and linear polynomial state equation were simultaneously applied to air^49^. For the other two materials, because the rock blasting process always involves a rapid increase in strain, the rock mass was modeled by the *MAT_PLASTIC_KINEMATIC constitutive model considering the strain rate effect^50^. Then, the *MAT_DRUCKER_PRAGER constitutive model was employed to depict the great deformation of the stemming plug under strong dynamic loads^51^. In addition to the material parameters of rocks from laboratory tests, the parameters of other materials were taken from previous studies and are provided in Tables 1, 2, 3, 4.

**Table 1:** Material parameters of the explosive.

*ρ*_e_ (kg m^−3^)	*D*_e_ (m s^−1^)	*A*_e_ (GPa)	*B*_e_ (GPa)	*R*_1_	*R*_2_
1100	3200	214	0.182	4.15	0.95

*ρ*_e_ is the desity. *D*_e_ is the detonation velocity; *A*_e_, *B*_e_, *R*_1_, and *R*_2_ are the parameters of state equation.

**Table 2:** Material parameters of air.

*ρ*_a_ (kg m^-3^)	*C*_0_–*C*_3_	*C*_4_	*C*_5_	*C*_6_
1.25	0.00	0.40	0.40	0.00

*ρ*_a_ is the desity. *C*_0_–*C*_6_ are the parameters of state equation.

**Table 3:** Material parameters of the rock mass.

*ρ*_r_ (kg m^−3^)	*E* (GPa)	Poisson ratio	*S*_c_ (MPa)	*S*_t_ (MPa)
2545	27.3	0.23	81.3	5.8

*ρ*_r_ is the desity. *E* is the elastic modulus. *S*_c_ and *S*_t_ are the compressive and tensile strengths.

**Table 4:** Material parameters of the stemming plug.

*ρ*_s_ (kg m^-3^)	*E*_s_ (MPa)	Poisson ratio	Cohesion (MPa)	*φ* (rad)
1850	20.0	0.28	0.18	0.56

*ρ*_s_ is the desity. *E*_s_ is the shear modulus. *φ* is the friction angle.

### Propagation of the blasting stress wave

**Figure 8:**
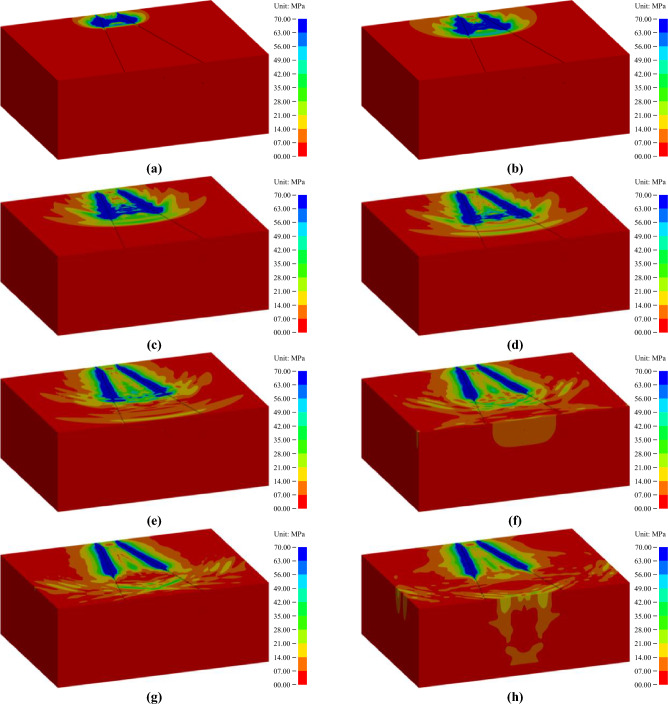
Propagation process of blasting stress waves under a conventional wedge cutting pattern: (**a**) 100 μs; (**b**) 200 μs; (**c**) 320 μs; (**d**) 420 μs; (**e**) 520 μs; (**f**) 660 μs; (**g**) 760 μs; and (**h**) 920 μs.

**Figure 9:**
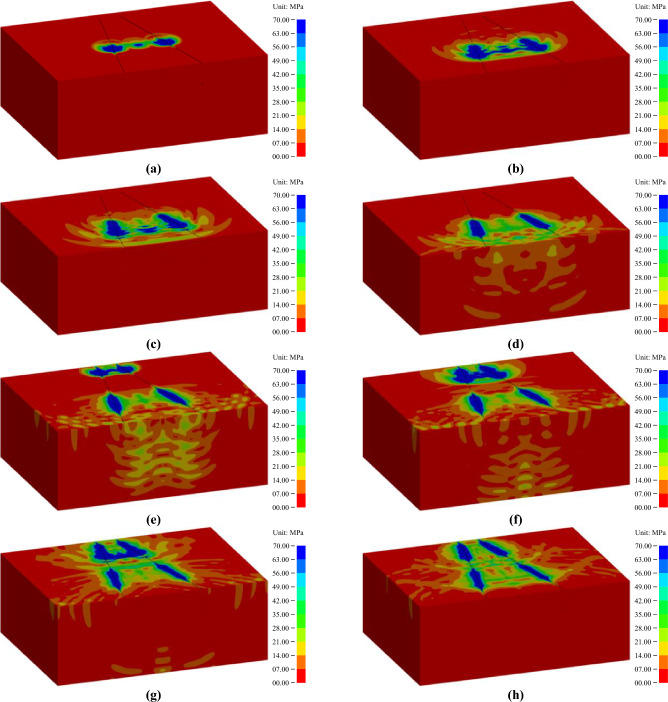
Propagation process of blasting stress waves under an innovative wedge cutting pattern: (a) 070 μs; (**b**) 160 μs; (**c**) 260 μs; (**d**) 430 μs; (**e**) 570 μs; (**f**) 660 μs; (**g**) 760 μs; and (**h**) 920 μs.

The finite element models containing the above information were obtained from the classical solution environment of LS-DYNA, and a dedicated postprocessor, namely, LS-PREPOST 4.5, was used to output the explicit dynamic simulation results. To clearly show the propagation of the stress wave, the whole-rock mass models were dissected along the horizontal symmetry section, and the lower part was reserved. Moreover, the direction of each rock mass model was also deliberately regulated according to the same angle. The propagation processes of the blasting stress wave under the two wedge cutting patterns are shown in Figs. 8 and 9, in which the stress strength decreased gradually from blue to red.

As displayed in Fig. 8, the detonation reaction of the blasting charge initiated from the bottom of each blasting hole, and an abundance of detonation energy was delivered into the rock mass to excite the stress wave. With the progress of the detonation reaction, the blasting stress wave also moved toward the heading face along the blasting holes. The blasting stress waves generated by each blasting hole can be superimposed on each other, and the superposition effect gradually weakened with increasing hole spacing. After the detonation reaction in each blasting hole was completed at 320 µs, the blasting stress wave continued to move toward the heading face. However, since no detonation reaction of the blasting charge continuously provides energy, the intensity of the blasting stress wave in the noncharge section was significantly weaker than that in the charge section. Conceivably, the weak stress wave cannot adequately destroy the upper rock mass, thus leading to the formation of large rock blocks in the upper section. When the blasting stress wave extended to the heading face at 520 µs, it was reflected as a tensile stress wave propagating in reverse. For most brittle materials, such as rocks and concrete, their tensile strength is usually less than 10% of their compressive strength; thus, the rock mass at the top region was vulnerable to tensile destruction. In addition, the newly generated tensile wave can be superimposed with the original compression wave to promote further destruction of the rock mass.

As illustrated in Fig. 9a–d, the detonation reaction of the upper charge was initiated at 0 µs, and the blasting stress wave was induced synchronously. With the development of the detonation reaction, the blasting stress wave propagated toward the heading face. At 160 µs, the detonation reaction in each blasting hole was completed, but the blasting stress wave continued to move toward the heading face. At 260 µs, after extending to the heading face, the stress wave was reflected as a tensile stress wave propagating in reverse, which can cause failure destruction of the rock mass near the heading face. Then, the newly induced tensile wave can also be superimposed with the original compression wave. The above analysis reveals that the blasting process of the upper charge involved a wedge cutting pattern with shallow holes. Moreover, because there was only a short distance of the uncharged section from the heading face to the upper charge, the rock mass in the upper uncharged section can be fully destroyed under the strong stress wave of the upper charge. Conceivably, the rock mass in the upper cutting cavity can be broken into small rock blocks that were easily removed.

Subsequently, as shown in Fig. 9e–h, the detonation reaction of the bottom charge initiated at 500 µs and produced a blasting stress wave. With the progress of the detonation reaction, the blasting stress wave developed toward the heading face. At 660 µs, the detonation reaction in each blasting hole was completed, but the blasting stress wave can move toward the heading face. Then, the stress wave of the bottom charge was superimposed on that of the upper charge to form a complex stress field. Due to the limitations of finite element simulation, the forming process of the cutting cavity cannot be obtained. In fact, upper charge blasting created a shallow cavity, thus providing a new free surface for bottom charge blasting. Therefore, the stress wave generated by the bottom charge is actually reflected as a tensile wave at the new free surface. Furthermore, the new free surface induced by the upper charge blasting can also significantly reduce the surrounding rock clamping action on the bottom rock mass. Similar to the previous description, due to the short distance of the uncharged section from the new surface to the bottom charge, the rock mass in the bottom uncharged section can also be fully destroyed under the strong stress wave of the bottom charge. Predictably, the rock mass in the bottom cutting cavity can be broken into small rock blocks that were easily ejected.

### Distribution features of blasting stress

**Figure 10:**
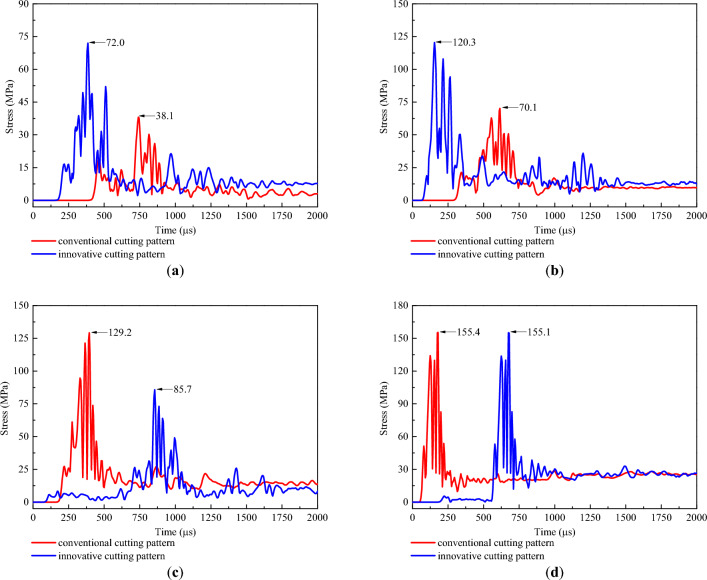
Stress‒time curves of typical measuring points on ML1: (**a**) 0.4 m; (**b**) 1.1 m; (**c**) 1.8 m; and (**d**) 2.5 m.

**Figure 11:**
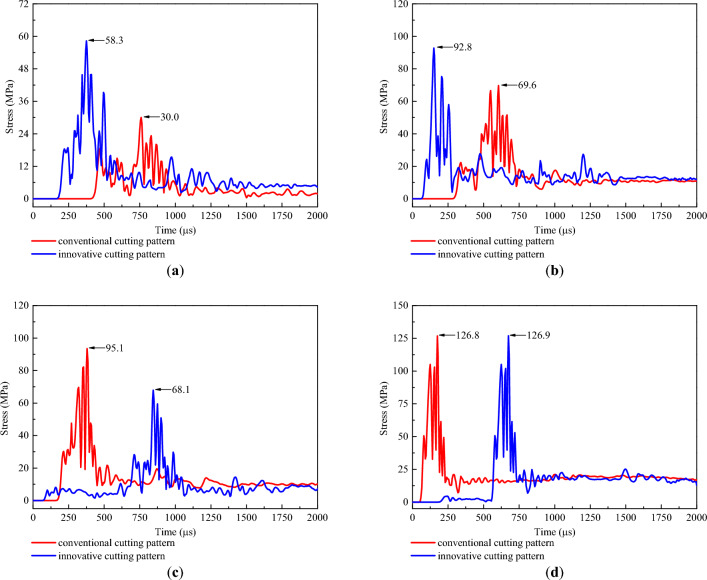
Stress‒time curves of typical measuring points on ML2: (**a**) 0.4 m; (**b**) 1.1 m; (**c**) 1.8 m; and (**d**) 2.5 m.

After understanding the propagation of the blasting stress wave, two representative measuring lines, ML1 and ML2, were installed in the rock mass model to reveal the stress distribution in the cutting cavity under different cutting patterns. The measuring lines ML1 and ML2 were both parallel to the vertical cutting holes and were fixed in the middle of the two vertical cutting holes and 200 mm above the upper vertical cutting hole, respectively, as indicated in Figs. 5 and 6. Moreover, a measuring point was arranged every 0.1 m along each measuring line. Then, the postprocessor LS-PREPOST4.5 was adopted to output the stress‒time curves of these measuring points, and the stress‒time curves of several typical measuring points on the two measuring lines are shown in Figs. 10 and 11.

According to Figs. 10 and 11, for the conventional cutting pattern, since the blasting stress wave started from the bottom cavity and propagated toward the heading face, the occurrence time of the peak stress was also gradually delayed from the bottom to the top. For the innovative cutting pattern, because the detonation time of the bottom charge was delayed compared with that of the upper charge, the occurrence times of peak stress at 0.4 m and 1.1 m were significantly earlier than those at 1.8 m and 2.5 m. This situation corresponded to the fact that wedge cutting blasting was divided into two blasting steps under the use of hole-inner delay. Furthermore, when the upper and bottom cutting cavities were studied separately, the occurrence time of peak stress at 1.1 m was earlier than that at 0.4 m, and the occurrence time of peak stress at 2.5 m was earlier than that at 1.8 m. This phenomenon reflected that the blasting stress waves propagating from bottom to top were generated in both the upper and bottom cavities, which corresponded to the propagation process of blasting stress waves stated previously.

**Figure 12:**
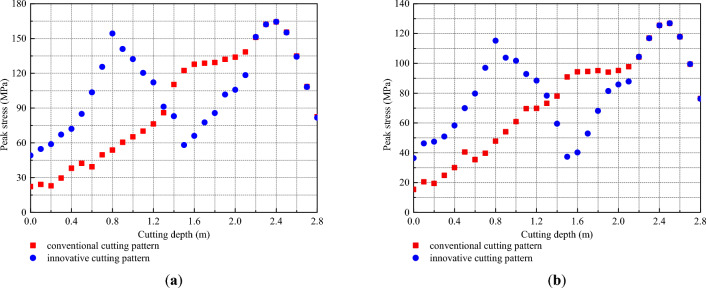
Variation curve of peak stress with cutting depth: (**a**) ML1 and (**b**) ML2.

To further reveal the stress distribution features, the peak stress of each measuring point was output to plot the variation curve of the peak stress with cutting depth, as presented in Fig. 12. For the conventional wedge cutting pattern, the peak stress first increased from 0.0 to 2.4 m and then decreased from 2.4 to 2.9 m with increasing cutting depth. At a cutting depth of 2.4 m, the peak stress reached a maximum value of 164.3 MPa. For the innovative wedge cutting pattern, in the range of 0.0–1.4 m from the upper cutting cavity, the peak stress first increased at 0.0–0.8 m, decreased at 0.8–1.4 m, and reached the first maximum value of 154.3 MPa at 0.8 m. In the range of 1.4–2.8 m from the bottom cutting cavity, the peak stress first increased at 1.4–2.4 m, then decreased at 2.4–2.8 m, and reached the second maximum value of 164.4 MPa at 2.4 m. As illustrated in Fig. 12b, for the conventional wedge cutting pattern, the peak stress first increased at 0.0–2.5 m and then decreased at 2.5–2.9 m with increasing cutting depth. At a cutting depth of 2.5 m, the peak stress reached a maximum value of 126.8 MPa. For the innovative wedge cutting pattern, in the range of 0.0–1.4 m from the upper cutting cavity, the peak stress first increased from 0.0 to 0.8 m, decreased from 0.8 to 1.4 m, and reached the first maximum value of 115.2 MPa at 0.8 m. In the range of 1.4–2.8 m from the bottom cutting cavity, the peak stress first increased from 1.4 to 2.5 m and then decreased from 2.5 to 2.8 m and reached the second maximum value of 126.9 MPa at 2.5 m. The variation trends of the peak stress in the upper and bottom cutting cavities were similar to those of the conventional wedge cutting pattern. Both upper charge blasting and bottom charge blasting can be regarded as conventional wedge cutting patterns using shallow blasting holes.

In addition, as shown in Fig. 12, the peak stress within 0.0–1.3 m under the innovative cutting pattern was greater than that under the conventional cutting pattern. This is because the blasting charge in the conventional cutting pattern was concentrated at the bottom section, and the innovative cutting pattern modified the blasting charge into the upper and bottom parts by using the hole-inner delay. The upward movement of the upper charge increased the stress strength in the upper cutting cavity. Then, the peak stress within 1.4–2.1 m under the innovative cutting pattern was less than that under the conventional cutting pattern, which was due to the lack of explosives in this section triggered by the upward movement of some explosives. Moreover, the peak stress within 2.2–2.8 m under the innovative cutting pattern was equal to that under the conventional cutting pattern. Through calculation, on measuring line ML1, the mean value of the peak stress within 0–1.4 m under the conventional cutting pattern was 52.7 MPa, and that under the innovative cutting pattern was 96.7 MPa. The latter was 1.83 times greater than the former. Moreover, on measuring line ML2, the mean value of the peak stress within 0–1.4 m under the conventional cutting pattern was 45.3 MPa, and that under the innovative cutting pattern was 75.0 MPa. The latter was 1.66 times greater than the former. It was observed that upper charge blasting can significantly increase the stress strength in the upper cutting cavity.

Based on the above discussion, for the conventional cutting pattern, a relatively low stress strength leads to insufficient destruction of the upper rock mass and the generation of large rock blocks in the upper cutting cavity, thus resulting in resistance to the expulsion of the bottom rock mass. For the innovative cutting pattern, the rock mass in the upper cavity is fully destroyed to form small rock blocks under relatively high stress strength, thus easily forming a shallow cutting cavity. Therefore, the resistance to the expulsion of the bottom rock mass was eliminated, and the newly generated free surface can reduce the clamping action exerted on the bottom rock mass. Due to the advantages of the first blasting of the upper charge, the rock mass in the bottom cutting cavity was easily destroyed and fully expelled. Then, a cutting cavity conforming to the design depth was be produced. Moreover, although the peak stress within 1.4–2.1 m under the innovative cutting pattern was less than that under the conventional cutting pattern, the rock mass in this range was relatively easy to destroy and expel due to the new free surface induced by the first blasting of the upper charge.

## Field application

### Project situation and blasting design

With the aim of thoroughly examining the practical use of wedge cutting patterns with hole-inner delay in deep-hole blasting driving construction, field applications were implemented in a rock tunnel at the Panji No. 3 Coal Mine in Huainan, China. Preliminary field surveys illustrated that the rock type of the underground tunnel was hard sandstone with a small amount of quartz. The basic performance parameters of sandstone were tested through laboratory experiments, and the specific results are listed in Table 3. The general implementation plan is as follows: with the same hole arrangement, full section blasting experiments under each cutting pattern were carried out several times, and then the overall blasting effect was evaluated and compared statistically and comprehensively. Prior to the current blasting experiments, the conventional cutting pattern had always been employed at the construction site. Thus, blasting designs using conventional and innovative cutting patterns were named the original and modified programs, respectively.

**Figure 13:**
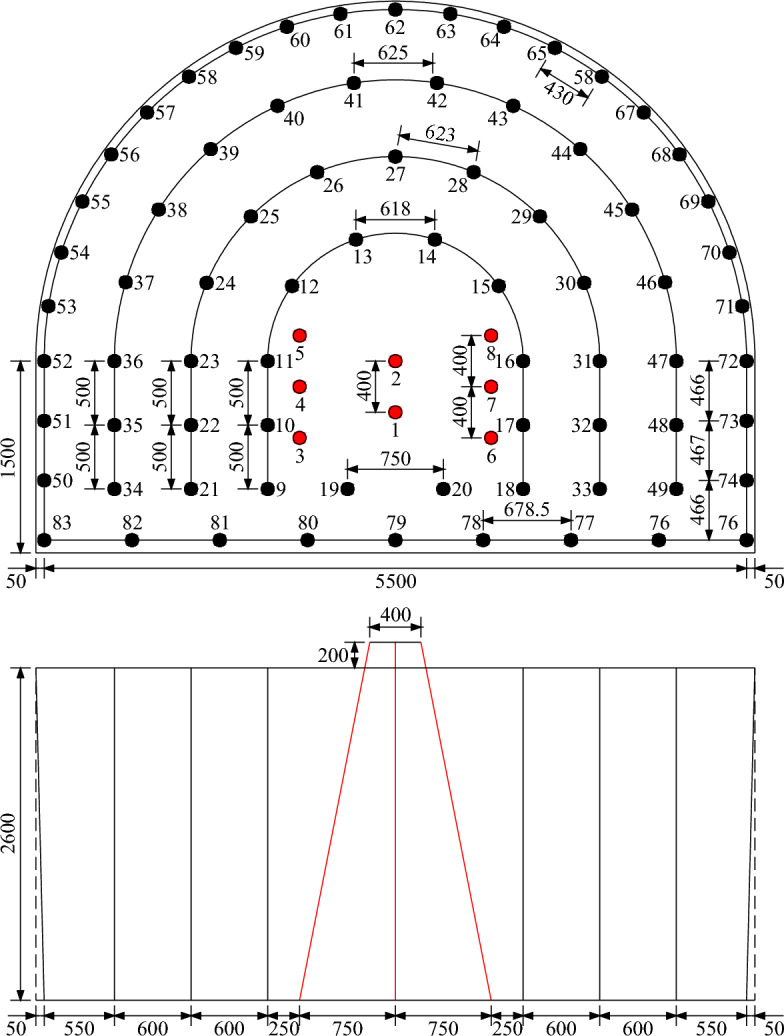
Diagram of the blasting hole arrangement (unit: mm).

During the field experiments, Φ 42 mm (Φ is the diameter of the blasting holes) blasting holes and explosive sticks with dimensions of *D* 29 mm × *L* 430 mm × *M* 310 g (*D*, *L*, and *M* are the diameter, length, and mass of the explosive sticks, respectively) were adopted in the profile holes, and Φ 42 mm blasting holes and explosive sticks with dimensions of *D* 35 mm × *L* 330 mm × *M* 350 g were employed in the other types of blasting holes. Large-diameter explosive sticks were used to increase the blasting load to ensure the breaking effect of the rock mass, and small-diameter explosive sticks were used to reduce the blasting load to control the peripheral smoothness of the tunnel contour. Moreover, the blasting materials and initiating equipment permitted by coal mines were water-glue explosives and electronic detonators. Since the innovative cutting pattern occupied an additional detonation segment, the five delay segments of 0, 30, 60, 90, and 120 ms in the original program were replaced with the six delay segments of 0, 30, 60, 80, 100, and 120 ms in the modified program. Due to the safety delay time of 130 ms specified in the Coal Mine Safety Regulations, a total delay time of 120 ms was still maintained. The specific arrangement of the blasting holes is shown in Fig. 13, and the blasting parameters under the two blasting programs are exhibited in Tables 5 and 6.

**Table 5:** Blasting parameters of the original program.

Hole type	Hole No	Hole amount	Detonator amount	Explosive per hole (kg)	Explosive subtotal (kg)	Detonation time (ms)
Cutting hole	1–8	8	8	1.40	11.20	0
Breaking hole	9–20	12	12	1.40	16.80	30
Breaking hole	21–33	13	13	1.40	18.20	60
Breaking hole	34–49	16	16	1.40	22.40	90
Profile hole	50–74	25	25	0.62	15.50	120
Lifter hole	75–83	9	9	1.05	9.45	120
Total	–	83	83	–	93.55	–

**Table 6:** Blasting parameters of the modified program.

Hole type	Hole no	Hole amount	Detonator amount	Explosive per hole (kg)	Explosive subtotal (kg)	Detonation time (ms)
Cutting hole	1–8	8	8	0.70	5.60	0 (upper)
Cutting hole	1–8		8	0.70	5.60	30 (bottom)
Breaking hole	9–20	12	12	1.40	16.80	60
Breaking hole	21–33	13	13	1.40	18.20	80
Breaking hole	34–49	16	16	1.40	22.40	100
Profile hole	50–74	25	25	0.62	15.50	120
Lifter hole	75–83	9	9	1.05	9.45	120
Total	–	83	91	–	93.55	–

### Results and Analysis

**Figure 14:**
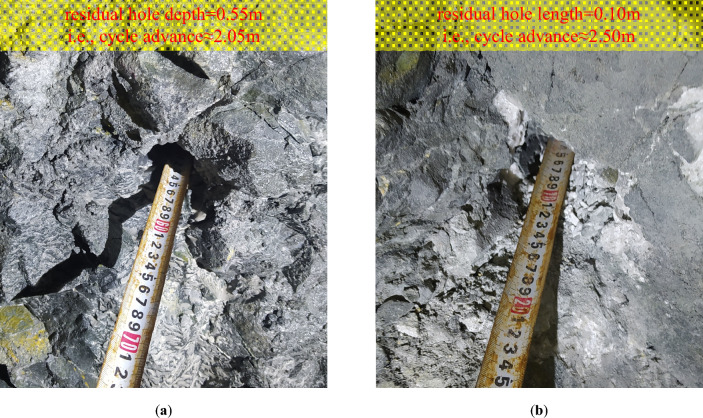
Measurement of the typical cycle advance at the blasting site: (**a**) Original program and (**b**) modified program.

According to the designed hole arrangement and blasting parameters, 10 full-section blasting experiments were conducted under each cutting pattern. Then, the cycle advance, hole utilization, specific explosive, and specific detonator were measured to indirectly reflect the cutting blasting efficiency. The latter three evaluation indices needed to be converted based on the cycle advance, and the measurement of the typical cycle advance is presented in Fig. 14. The statistics of the overall blasting effects under the original and modified programs are given in Tables 7 and 8.

**Table 7:** Statistics of overall blasting effects under the original program.

No	Cycle advance (m)	Hole utilization (%)	Specific explosive (kg m^−3^)	Specific detonator (PCS m^−3^)
1	2.05	78.8	2.20	1.95
2	2.00	76.9	2.26	2.00
3	2.05	78.8	2.20	1.95
4	2.00	76.9	2.26	2.00
5	1.95	75.0	2.32	2.05
6	2.05	78.8	2.20	1.95
7	2.10	80.8	2.15	1.91
8	1.95	75.0	2.32	2.05
9	2.05	78.8	2.20	1.95
10	2.00	76.9	2.26	2.00

**Table 8:** Statistics of overall blasting effects under the modified program.

No	Cycle advance (m)	Hole utilization (%)	Specific explosive (kg m^−3^)	Specific detonator (PCS m^−3^)
1	2.45	94.2	1.84	1.79
2	2.50	96.2	1.81	1.76
3	2.50	96.2	1.81	1.76
4	2.55	98.1	1.77	1.72
5	2.50	96.2	1.81	1.76
6	2.45	94.2	1.84	1.79
7	2.55	98.1	1.77	1.72
8	2.45	94.2	1.84	1.79
9	2.50	96.2	1.81	1.76
10	2.45	94.2	1.84	1.79

According to Table 7, under the conventional wedge cutting pattern, the mean values of the four evaluation indices of rock tunnel driving were 2.02 m, 77.7%, 2.24 kg m^−3^, and 1.98 PCS m^−3^. From Table 8, using the innovative wedge cutting pattern, the mean values of the four evaluation indices reached 2.49 m, 95.8%, 1.81 kg m^−3^, and 1.76 PCS m^−3^. Clearly, in contrast to the conventional cutting pattern, the innovative cutting pattern increased the mean values of the four evaluation indices by 0.47 m, 18.1%,–0.43 kg m^−3^ and–0.22 PCS m^−3^. Together, the increase in driving speed and decrease in material expenditure strongly confirmed that the wedge cutting blasting technique with hole-inner delay was applicable to deep-hole blasting driving of rock tunnels.

## Conclusion

In the present paper, the mechanisms of rock failure and cavity formation of the innovative wedge cutting blasting pattern and its practical application effect were investigated thoroughly via theoretical analysis, finite element simulation, and field experiments, and several useful research findings are summarized as follows:

1. Theoretical analyses indicated that the conventional wedge cutting pattern cannot achieve a satisfactory effect in deep-hole blasting under the resistance from large upper rock blocks and the clamping action from the surrounding rock. However, via the innovative wedge cutting pattern, the first blasting of the upper charge can effectively break the upper rock mass and create a new free surface, thus reducing the above difficulties for the subsequent delayed blasting of the bottom charge.
2. The propagation of blasting stress waves under the two cutting patterns was visually shown via finite element simulation. Importantly, the first blasting of the upper charge remarkably increased the stress strength in the upper cutting cavity by 66–83%, which was beneficial for the breaking of the upper rock mass and the generation of a new free surface. Then, the rock mass in the bottom cutting cavity was broken up and ejected easily under the delayed blasting of the bottom charge, thus inducing a cutting cavity conforming to the design depth.
3. In contrast to the conventional cutting pattern, the proposed innovative cutting pattern can realize a significant increase in cycle advance and hole utilization and a negative growth in a specific explosive and specific detonator. Field application results strongly substantiated that the wedge cutting blasting technique with hole-inner delay assumed excellent applicability to deep-hole blasting driving of underground rock tunnels.

## Data Availability

All the data generated or analyzed during the current study are included in this published article.
